# Australian and New Zealand Medical Students’ Attitudes and Confidence towards Providing Nutrition Care in Practice

**DOI:** 10.3390/nu12030598

**Published:** 2020-02-25

**Authors:** Breanna Lepre, Jennifer Crowley, Dineo Mpe, Harsh Bhoopatkar, Kylie J. Mansfield, Clare Wall, Eleanor J. Beck

**Affiliations:** 1 School of Medicine, University of Wollongong, and Illawarra Health & Medical Research Institute, Northfields Ave, Wollongong, NSW 2522, Australia; kylie@uow.edu.au (K.J.M.); eleanor@uow.edu.au (E.J.B.); 2 School of Medical Sciences, Faculty of Medical and Health Sciences, The University of Auckland, Private Bag 92019, Auckland 1142, New Zealand; j.crowley@auckland.ac.nz (J.C.); d.mpe@auckland.ac.nz (D.M.); h.bhoopatkar@auckland.ac.nz (H.B.); c.wall@auckland.ac.nz (C.W.)

**Keywords:** doctors, medical education, medical students, nutrition care, nutrition education

## Abstract

The prevalence of lifestyle-related chronic disease is increasing. Doctors in primary care are ideally placed to support patient nutrition care, but recent reviews show education is still lacking. This study aimed to identify medical students’ attitudes towards the role of nutrition in health, nutrition knowledge, and perceptions of nutrition education, in postgraduate (Australia) and undergraduate (New Zealand) programs in order to identify gaps in nutrition knowledge and skills to better inform future education. Second-year graduate and third-year undergraduate students participated in semi-structured focus groups and interviews. A general inductive approach was used to investigate students’ (1) attitudes toward the role of nutrition in health, (2) nutrition knowledge based on nutrition-specific competencies and (3) perceived adequacy of nutrition education received. Interviews (nine) and focus groups (seven) identified four common themes: (1) role of medical practitioners in nutrition care, (2) barriers to nutrition education, (3) nutrition knowledge, and (4) nutrition-related skills. Students perceive that doctors are well-placed to provide some level of nutrition care, but poor translation of nutrition knowledge to clinical contexts is a key limitation in nutrition education. In summary, nutrition education may be insufficient to support the nutrition-related competency development of the undergraduate and postgraduate student participants in this study. Focusing on the integration of these skills into the curriculum may be a priority.

## 1. Introduction

The prevalence of lifestyle-related chronic disease is increasing worldwide [1]. The World Health Organisation (WHO) predicts that, by 2020, chronic diseases will account for almost three quarters of all deaths worldwide [2]. Chronic diseases include obesity, diabetes, cardiovascular diseases, cancer and osteoporosis, for which poor nutrition is a risk factor [3]. Chronic disease imposes immense social and economic burdens, associated with healthcare costs and lost productivity related to illness and death [3]. In Australia, an estimated AU$27 billion is spent annually on chronic disease, or 36% of total health expenditure [1]. In New Zealand (NZ), obesity alone is responsible for an estimated 4.4% of the total health expenditure [4]. In response to the economic burden associated with chronic disease there is demand for changes in health budgets, the capacity of health systems and the competency of the health workforce [5]. Improving long-term nutrition behaviour is a cost-effective tool in the prevention and management of chronic disease [6].

Doctors who work in primary care are often a patient’s first point of contact with the healthcare system [7,8,9], making them ideally placed to support patient nutrition care. Nutrition care refers to any care that aims to improve the nutrition behaviours and subsequent health outcomes of an individual [10]. Primary care doctors require nutrition knowledge and skills to enable them to provide nutrition care to patients, or to identify patients for whom specialist dietetic advice is required. The international literature suggests that doctors lack nutrition knowledge and skills to provide high quality nutrition care [11,12,13]. The barriers that doctors face in providing nutrition care include a lack of time and reimbursement, insufficient resources, inadequate nutrition education and limited confidence to deliver this care [14]. In past decades, there have been numerous articles [15,16], symposia [17,18] and congressional hearings [19] dedicated to the need to improve medical nutrition training. However, in the most recent synthesis of the literature on medical nutrition education, it is reported that nutrition remains insufficient in medical education, regardless of location, setting or stage of medical education [20]. Furthermore, it is reported that that deficits in medical students’ knowledge, skills and a lack of confidence to provide nutrition counselling are likely to continue into clinical practice.

In Australia and NZ (ANZ), accreditation standards for medical education are set by the Australian Medical Council (AMC), and informed by AMC curriculum guidelines [21]. Despite the potential of doctors in providing nutrition care, the AMC framework does not include nutrition-specific competencies, although broad competencies related to chronic disease, public health and general clinical and professional skills are included [21]. To address the lack of nutrition competencies in ANZ medical education, a Nutrition Competency Framework (NCF) was previously developed, which describes four knowledge and five skill-based nutrition competencies mapped to the AMC Graduate Outcome Statements [21,22]. The nutrition competencies are presented in Table 1.

Limited literature exists on nutrition education provided to medical students in ANZ and investigation to support improvements in medical nutrition training are warranted. The aims of this study were: (1) to identify undergraduate and postgraduate medical students’ attitudes to the role of nutrition in health, nutrition knowledge based on NCF, and their perceptions of nutrition education, in a postgraduate (Australia) and undergraduate program (NZ); (2) to identify gaps in students’ nutrition knowledge and skills to inform opportunities for nutrition education.

## 2. Materials and Methods

A qualitative study design was used to produce thematic descriptions of nutrition education received by medical students. Focus groups, face-to-face and phone interviews were conducted with second-year students enrolled in an Australian postgraduate Doctor of Medicine (MD) programme and focus groups were conducted with third-year students enrolled in a Bachelor of Medicine and Bachelor of Surgery (MBCHB) programme in NZ. University of Wollongong (UoW) students were in the second year of a four-year degree and University of Auckland (UoA) students were in the third year of a six-year degree, i.e., both groups were in their final preclinical year. Facilitators provided a non-experimental, non-threatening environment to allow participants to speak freely using guided questions (Table 2) to achieve the study aims. Following poor student participation in the UoW focus groups, it was determined to include one-to-one semi-structured interviews to better suit students’ schedules. Ethics approval was received from the University of Wollongong Human Research Ethics Committee (2018/137) and University of Auckland Human Participants Ethics Committee (protocol number 021298).

### Recruitment

An information leaflet was distributed to relevant students using university emails and class representatives. Students who volunteered to participate in the study were contacted via email by their respective student investigator. Exclusion criteria included students who had previously undertaken a nutrition or medical degree. Recruitment was confirmed by students’ completion of a consent form.

Open-ended questions were used to guide focus groups sessions and interviews. Questions were developed by the research team at UoW and UoA using an inquiry logic approach based on nutrition competencies outlined in the NCF. Questions were piloted with one individual and one group (4 medical students) and question order was modified to improve sequencing. Data collection continued until data saturation was achieved (UoA), namely, until data did not reveal new information, or until the university session ended (UoW) [23]. Focus group and interview sessions were digitally recorded and transcribed verbatim. All data was de-identified to protect participant confidentiality.

A general inductive approach was used to analyse the data. At both sites, data coding and analysis was completed manually by two researchers (one student and one supervisor) to ensure rigour in analysis. Codes and themes were used to categorise data and to make intergroup comparisons.

Data analysis was guided by grounded theory [24]. Researchers detailed semantics in relation to questions about nutrition knowledge but used an inductive approach to explore students’ attitudes towards nutrition knowledge. Firstly, open manual coding of all discussions was conducted to identify recurring themes. Codes were then summarised into relevant themes for each participant/group. Sub-categories were identified within major themes to differentiate participants’ responses. After independently coding the data, the two researchers compared and discussed their respective findings. Any discrepancies were discussed until a consensus was reached. A schematic analysis was applied to dominant themes to identify the impact of nutrition education in medical curricula on students’ perceptions of nutrition care in practice and suggestions for improvement.

## 3. Results

One face-to-face focus group (n = 4) and nine individual face-to-face and phone interviews were conducted (n = 13) from ninety-one students in year two of the postgraduate MD programme (response rate (RR) 14%). Thirty-five year three medical students (n = 35) from a potential pool of two hundred and eighty-three undergraduate students undertaking the MBChB programme at UoA (RR 12.3%) participated in six focus groups. Each undergraduate focus group had between five and eight participants and all focus groups lasted between forty-five and eighty minutes. The majority of participants were female (n = 26, 54%) and were from an undergraduate medical program (n = 25, 52%).

Analysis of the combined data resulted in four common interrelated themes: (1) the role of doctors in providing nutrition care, (2) nutrition knowledge, (3) nutrition-related skills and (4) medical nutrition education. Two additional themes emerged, one from the postgraduate participants: (5) personal beliefs shaping attitudes toward nutrition and one from the undergraduate participants: (6) nutrition for population health.

### 3.1. Theme 1 – The Role of the Doctor in Providing Nutrition Care

#### 3.1.1. Doctors Have a Role in Providing Nutrition Care

Overall, participants agreed that doctors have a role in providing nutrition care to patients, including prevention and treatment of lifestyle-related disease. Indicative quotes from transcripts are provided to illustrate key themes identified from the data.

“I definitely think that, especially with the obesity epidemic, that more nutritional advice is warranted, to try and prevent a lot of the diseases.”

(P10, UoW)

“Nutrition is a big part of health… It would be silly not to be responsible for the element if you are treating someone.”

(P2, FG2, UoA)

#### 3.1.2. Doctors Are Well-Placed to Provide Nutrition Care 

Participants felt that doctors, especially general practitioners (GPs) were best placed to provide nutrition care. However, many participants identified limits to their scope of practice and considered the primary role of doctors to be complementary to that of nutritionists/dietitians, who participants believe are able to provide more personalised and ongoing nutrition care.

“GPs are really well placed to start that [nutrition] conversation with patients and take it as far as they can. And then if patients want a lot of personalised, ongoing information then it might be better off referring to a nutritionist.”

(P8, UoW)

“If you are in a situation where a patient asked you for advice, I think we should still be able to give something… We need sufficient knowledge to give some advice… It won’t be at the same level as dietitians or nutritionists.”

(P4, FG2, UoA)

#### 3.1.3. GPs Are an Accessible Source of Nutrition Care

Some participants perceived that GPs should be more involved in nutrition care, as they (GPs) were seen as an accessible source of nutrition care.

“In [the] primary care setting not everyone has access to dietitians, so I think also GP’s would be equipped with that kind of knowledge as well.”

(P2, FG3, UoA)

### 3.2. Theme 2 – Knowledge-Based Nutrition Confidence

#### 3.2.1. Participants Had Variable Nutrition Knowledge 

In response to knowledge-based questions, participants demonstrated variable nutrition knowledge. Collectively, most participants were able to identify the relevant principles and concepts of a healthy diet, such as quantity, variety and balance.

“I suppose a balance between the macro-nutrition and micro-nutrition, so a balance between protein, carbohydrate, fats, as well as well-balanced in terms of the vitamins that are required would be a general answer.”

(P10, UoW)

Overall, participants acknowledged the link between diet and the development of disease and that nutrition is important in the prevention of lifestyle-related disease. The majority of participants were able to identify common nutrition-related causes of mortality and morbidity in the population.

“Most of the diseases that are a problem today are nutrition-related diseases”

(P1, FG2, UoA)

#### 3.2.2. Dietary Management Strategies for the Prevention and Treatment of Chronic Disease

When prompted, most participants were unable to describe dietary management strategies for prevention and treatment of chronic disease. Most students attributed this to the nutrition education received thus far, which focused mainly on biomedical mechanisms and pharmacology of disease rather than lifestyle interventions.

“It’s [nutrition] mentioned and then it’s like ‘and this is the drug you put them on’.”

(P3, UoW)

“It’s one thing saying “you need to lose weight” but it is another to give them the strategies to do that”

(P4, FG2, UoA)

### 3.3. Theme 3: Skill-Based Nutrition Confidence

#### 3.3.1. Lack of Confidence in the Application of Skill-Based Nutrition Competencies 

Most participants were not confident in skill-based nutrition competencies within the Nutrition Competency Framework (NCF).

“Uh, I could grope my way towards it, but I wouldn’t feel very confident in doing it [nutrition care].”

(P6, UoW)

“I think we have had enough so far if it were then to be applied…. and shown how it is used in clinical practice later.”

(P2, FG2, UoA)

To assess their confidence in the application of evidence-based approaches to nutrition care, participants were asked how to locate resources related to patient nutritional management. Generally, participants were unable to list evidence-based health resources, such as the Australian Guide to Healthy Eating [25]; however, when prompted, some participants suggested professional bodies, such as Diabetes Australia.

“I think ones for specific(s) like Diabetes Australia does one for diabetes, and… Coeliac Australia does one for coeliac diets and there’s all these ones for FODMAPS as well that are available through those kinds of organisations.”

(P4, UoW)

Participants differed in their perceived ability to discern between accurate and inaccurate nutrition information.

“…There are so many sources of information that it can almost be hard to locate where you got the information from because it just seems like we are constantly surrounded by it….”

(P2, FG3, UoA)

#### 3.3.2. A Multidisciplinary Approach to Nutrition Care 

Collectively, all participants, agreed that a multidisciplinary approach to nutrition assessment and management can be effective for the delivery of high-quality nutrition care. Most participants felt they would readily refer to or consult with another health practitioner for the nutritional management of patients.

“I suppose I’d be happy to give very basic advice, but beyond that I’d probably be inclined to refer onward if I thought that they needed a proper nutritional assessment.”

(P10, UoW)

“I think that [Doctors] have to have… a base knowledge, up to date and current and have to be able to describe the basis of a healthy diet and a bad diet. But when it comes down to the… finer details and the specialised diets for different things, then they should give a referral to nutritionists.”

(P5, FG5, UoA)

### 3.4. Theme 4 – Medical Nutrition Education

All participants agreed that doctors have a role in providing basic nutrition care to patients yet, based on their current nutrition education, the majority of participants did not feel prepared to provide dietary advice. Participants identified limitations and suggestions to improve their nutrition education.

#### 3.4.1. Poor Translation of Nutrition Science to Clinical Application 

Collectively, participants perceived that there was poor application of the basic sciences underpinning nutrition in clinical contexts. Also, many participants noted difficulty in translating nutrition science to food-based recommendations.

“We have been taught functions for these vitamins and this mineral… I would struggle to see how it would translate to patients.”

(P3, FG2, UoA)

“In terms of the curriculum, I don’t think we are given enough information about the practical aspects of nutrition.”

(P13, UoW)

#### 3.4.2. Poor Integration of Nutrition into Medical Education 

Overall, participants felt that nutrition education was relatively limited and marginalised, as it was not integrated into all programme modules.

“It (nutrition education) needs to be more integrated into other modules”

(P3, FG5, UoA)

“It seemed to me that our framework was that it [nutrition education] all happened in the same fortnight… So, the foundation was put there to really build on it, but we never got the chance.”

(P3, UoW)

In contrast, some participants perceived that the nutrition education received was appropriate for pre-clinical medical education, with a caveat that more nutrition education should be provided in the future. Other participants perceived that despite shortcomings in their nutrition curriculum, the lack of time and space in the medical curriculum would make it difficult to increase nutrition teaching hours.

“I think… there are a lot of things that I wouldn’t be able to do a management plan for. We are not expected to know how to completely intervene for certain things at this stage.”

(P2, FG3, UoA)

“I understand that in our first year and a half the amount of curriculum is so much that adding to that may not be welcome or feasible, and even necessary at this stage of training.”

(P12, UoW)

#### 3.4.3. Poor Engagement with Nutrition Professionals 

Most postgraduate participants identified limited exposure to other clinicians, including nutrition experts, as a barrier to providing nutrition care. Collectively, both participant groups recommended more interprofessional approaches to nutrition education to consolidate learning. Specifically, participants suggested that increased engagement with dietitians in small group activities would be beneficial, to demonstrate the application of nutrition care and to foster nutrition-related skills development [26].

“Small group activities are quite a good place to discuss nutrition.”

(P5, FG2, UoA)

“I feel like there either needs to be more from those sorts of clinicians that come in or at least (some) emphasise to know where your limitations are and refer onwards and even say ‘look, do A, B and C, but once it goes beyond there refer to a dietitian’.”

(P10, UoW)

### 3.5. Theme 5 – Personal Beliefs Shaping Students’ Attitudes Towards Nutrition

#### Personal Experience of Specific Dietary Advice 

Postgraduate participants identified that the lack of faculty to provide nutrition education influenced their attitude to nutrition care. Postgraduate participants also perceived that their personal experience of adherence to specific dietary advice was acceptable nutrition care. Undergraduate participants were taught by a dietitian and did not share this view.

“Another good thing that I’ve read is not buying anything with ingredients that a third grader couldn’t read”

(P7, UoW)

Graduate participants’ beliefs about food and nutrition influenced their perceptions of relevant nutrition knowledge required in training, regardless of whether or not this advice might be considered appropriate by those with nutrition expertise.

“I guess the information is out there, and when we get guidelines on what a ketosis diet is, and what a plant-based diet is, then we’ll [students] be better off”

(P1, UoW)

### 3.6. Theme 6 – Nutrition for Population Health

#### 3.6.1. Environmental Determinants as Limitations to Dietary Adherence

Undergraduate participants identified environmental determinants, such as socioeconomic status, geographic location and health literacy as limitations in patients’ abilities to change nutrition behaviours. Some participants identified different population groups, such as the elderly, as vulnerable to food insecurity and inequitable access to healthy food in NZ.

“I think also the elderly, they can’t take care of themselves yep, they are more prone to problems.”

(P3, FG3, UoA)

“In New Zealand especially, there is a lot higher prevalence of … fast food and … low nutritional but cheap restaurants related to low socioeconomic gradients.”

(P3, FG4, UoA)

#### 3.6.2. Time Is a Limitation to the Provision of Nutrition Care

Limitations within the healthcare system, such as short consultation times, were also identified as a barrier to providing nutrition care in practice.

“Nutrition is more of an attitude rather than a set of guidelines and sharing that attitude is what shares… healthy beliefs… sharing attitude I feel is much more complex… how do we share that with a patient when you might only have like 10–15 min?”

(P3, FG2, UoA)

#### 3.6.3. The Role of Policy and Media in Health 

Participants perceived that doctors’ efforts to drive dietary change needed to be matched with support from other sectors, including government policy and health promotion initiatives from non-governmental agencies, such as the Cancer Council Australia [27]. These perceptions came with the recognition that media, particularly social media, has influence in the psychology of health.

“I don’t believe that having better educated doctors is going to be enough. It needs to be a public health message and I feel like nutrition and dietitians should be publicly funded.”

(P1, FG5, UoA)

## 4. Discussion

This study investigated ANZ medical students’ attitudes toward the role of nutrition in health, nutrition knowledge and perceptions of nutrition education received in their respective institutions. The results overall support the existing literature on the lack of nutrition education in medical programs [20]. This is not related to students’ interest in nutrition and, in fact, participants felt that doctors, particularly GPs, were well-placed to provide nutrition care and have an important role in providing this care. Participants identified barriers to providing nutrition care in practice, which included nutrition-related self-efficacy and locating reputable nutrition information. Participants reported limitations to their current nutrition education, including the poor application of nutrition science to clinical contexts and limited engagement with other health professionals. Some graduate participants expressed varying levels of nutrition knowledge based on personal beliefs, which were perceived as appropriate nutrition care. Cultural and environmental determinants of health were also recognised a limitation to changing patients’ nutrition behaviour.

Medical students’ understanding that doctors have an important role in patient nutrition care echoes the existing international literature related to medical students, doctors and medical educators [7,28]. Despite students’ positive attitudes toward the importance of nutrition care in this study, the current level of dietary advice provided by medical practitioners appears to be infrequent and highly variable [29]. In a survey of Australian GPs, it was reported that most (72%) felt it was their responsibility to provide nutrition counselling yet, nutrition counselling occurred in only 30% of patient encounters for chronic illness, 25% of visits for cardiovascular disease, 31% of visits for hypertension, 45% of visits for diabetes mellitus and 33% of visits by obese patients (body mass index >30 kg m^−2^), for all of which nutrition is part of the aetiology and management of the aforementioned conditions [30]. It appears that students’ perceptions of the health workforce do not match the experience of recent graduates. This may be related to a lack of nutrition knowledge and perceived confidence in providing nutrition care. When doctors lack nutrition knowledge and the confidence to provide nutrition care, they are unlikely to provide nutrition care as often as would be ideal, or to recognise when patients would benefit from a referral to a dietitian [31].

Competence in skills refers to the ability to execute a task [32]. In lieu of competence in nutrition care not being assessed in ANZ medical education, participants reported their perceptions of confidence to provide nutrition care. In the current study, participants displayed a perceived greater confidence in answering knowledge-based questions, which may be related to their stage in medical education. Whilst knowledge-based competencies are necessary to interpret new concepts in nutrition, skill-based competencies are essential for the application of nutrition knowledge in practice [33]. In a recent NZ study, graduating medical students reported low nutrition-related self-efficacy and perceived greater confidence discussing the role of nutrition in health and disease than aspects of nutrition counselling [34]. In another NZ study, graduating medical students, GP registrars and GPs’ attitudes toward nutrition care and self-perceived skills in providing nutrition care in practice were compared [35]. General practitioners displayed a perceived greater confidence in facilitating nutrition care than students or GP registrars, although the difference, while not significant, was attributed to GPs’ greater experience. All three groups of doctors reported moderate confidence in providing nutrition care, which suggests that, post-training, medical students are unlikely to fill gaps in their nutrition knowledge [35]. Therefore, it is important for students to have nutrition knowledge and witness the provision of nutrition care in total patient care. Students in the present study suggested increasing nutrition content across organ-based modules to emphasise the relevance of nutrition in total patient care and to avoid conflict in an already crowded curriculum. Evidence exists that vertical and horizontal integration of nutrition content across the learning continuum improves students’ nutrition-related competency development and can be assessed by objective structured clinical examination (OSCE) scores [36]. Students perceived that engagement with dietitians in small group activities would improve their confidence in dietary counselling and awareness of the safe scope of nutrition practice. Evidence exists that interdisciplinary approaches are more effective in improving nutrition education in the medical curriculum [37]. This suggests that dietitians may have a role in the planning and development of medical education to drive the implementation of nutrition competencies into medical curricula.

The questions and inquiry logic for this study were designed to explore students’ perceptions of their nutrition-related knowledge and skills. Many participants from the postgraduate programme indicated that their nutrition knowledge was relatively self-focused. Schwartz et al. (1991) reported that medical internists’ use of preventative interventions is associated with habit, attitude and a lack of adequate knowledge [38]. Perlstein et al. (2016) compared students’ knowledge of Australian dietary recommendations to the dietary practices of first-year medical students and reported a disconnect between students’ nutrition knowledge and their own dietary behaviours [39]. This is particularly concerning, given that a lack of nutrition knowledge influences attitudes towards and provision of nutrition care [40]. The Cooking for Health Optimization with Patients (CHOP) programme is an innovative approach to medical nutrition education, whereby first and second-year medical students undertake elective, integrated learning modules in evidence-based nutrition science and culinary skills for chronic disease management [41]. The intervention is reported to be more effective than traditional nutrition education in improving students’ dietary behaviours and competency in nutrition counselling [41]. While medical students should be taught evidence-based practices which scaffold all areas of medicine, formal adoption of the NCF or a similar model seems necessary to increase students’ preparation in nutrition during their medical education. This, and more personalised and engaging approaches to nutrition education, such as the CHOP programme, are likely to improve students’ nutrition knowledge and increase their confidence in skills for nutrition care [42]. More importantly, medical graduates may be more diligent in providing evidence-based information when providing nutrition care.

The time allocated to nutrition in medical curricula, how nutrition is taught and by whom, varies between medical programmes, based on the judgement of the relevant faculty, who have autonomy in determining how competencies are represented in medical education and assessment [43]. Students in the current study highlight this, identifying that, if doctors, faculty and curriculum planners consider nutrition education important, ways would be found to include adequate nutrition training throughout all stages of medical education [44]. Therefore, mandating nutrition content through competency frameworks, including assessment, is crucial, to provide opportunities to scaffold learning throughout medical education to maximise the acquisition of nutrition knowledge and skills. Currently, the NCF provides a reference point from which universities can integrate nutrition-related competencies into existing medical curricula. Given that accreditation standards act as a framework for curriculum content, the inclusion of nutrition competencies in accreditation standards would provide an incentive for ANZ universities to prioritise medical nutrition education.

This study adds depth and greater understanding of the scope of nutrition education medical students may receive in ANZ. The response rates, of less than 15%, and sampling methods used in this study are likely to have recruited participants with an interest in nutrition and/or nutrition education, which potentially introduces a sampling bias. The low response rates in both groups may be due to the high workload of students, the voluntary nature of the survey or the importance placed upon contributing to evaluations. Thirdly, qualitative research using focus groups and interviews does not provide an objective method for assessing nutrition knowledge, but does provide insights into data that quantitative methods may not provide [45]. The use of the same focus group facilitator in each respective location ensured rigour in focus group design [45]. Lastly, this study was limited to only two universities and the generalisability of these results should be cautioned.

## 5. Conclusions

Medical students perceived that doctors are well-placed to provide some level of nutrition care to patients. However, poor translation of nutrition knowledge into the clinical context is a key limitation in nutrition education. A more interprofessional approach to nutrition education, whereby nutrition professionals model the delivery of nutrition care, is suggested. Competency frameworks, such as the NCF, may be utilised with vertical and horizontal integration of nutrition into existing medical curricula. It would seem timely to further promote nutrition care with further investigation required to establish the best practices to benefit future medical nutrition education in ANZ.

## Figures and Tables

**Table 1:** Knowledge and skill-based nutrition competencies in The Nutrition Competency Framework.

**Knowledge-based Nutrition Competencies**
K1 – Sciences: Demonstrate understanding of the basic sciences in relation to nutrition
K2 – Prevention: Demonstrate knowledge of the interactive role of nutrition in health and the prevention of disease
K3 – Treatment: Demonstrate knowledge of the evidence-based dietary strategies for prevention and treatment of disease
K4 – Food: Demonstrate awareness of food, sources of nutrients, food habits and the cultural and social importance of food
**Skill-based Nutrition Competencies**
S1 – Risk: Demonstrate skills in the identification of nutritional risk, nutritional deficits and excesses
S2 – Critical: Demonstrate ability to interpret nutrition evidence in a critical and a scientific manner and apply appropriately in clinical practice
S3 – Application: Demonstrate ability to apply basic dietary strategies for prevention and treatment of medical conditions, disease and trauma, with recognition that many nutritional issues require specialist management by a dietitian
S4 – Ethics: Demonstrate the ability to apply principles of ethics related to nutritional management
S5 – Team: Demonstrate ability to work effectively in a team with other health professionals to deliver optimal nutrition care

**Table 2:** Interview guide and inquiry logic.

Interview Questions	Inquiry Logic
Do you think doctors have a role in providing nutrition information to patients?	Explore students’ perceptions of their role in providing nutrition care.
On a scale of 1–5 (1 being poor and 5 being excellent), please rate the nutrition training you have received so far in your medical education. (Probe) - How can this be improved?	Identify students’ perceptions of their nutrition training and ways it can be improved.
Can you identify what constitutes a healthy diet?	Identify students’ perceptions of a healthy diet.
Do you think nutrition is important in preventing lifestyle diseases? (Probe) - Identify lifestyle causes of morbidity and mortality - Identify management strategies for these diseases	Identify students’ perceptions of the link between nutrition and the development of lifestyle-related disease.
Can you tell me about diseases and medical conditions that affect nutrition requirements? (Probe) - Different populations whose dietary intake and requirements may differ from the average population	Identify students’ perceptions of knowledge of medical conditions that affect nutrition requirements.
Can you tell me about treatments/ medications that may alter a patient’s nutrition intake and/or requirements?	Identify students’ knowledge of medical treatments/medications that affect nutritional status.
Can you tell me about the psychological and societal causes of malnutrition? (Probe) - What risk factors in a patient would alert you to a risk of malnutrition?	Explore students’ perceptions of the psychological and societal causes of malnutrition.
Do you think that healthy food is accessible in New Zealand/Australia? (Probe) - Is the access equitable? - Who is most vulnerable to food insecurity?	Explore students’ perceptions of the accessibility of healthy food in New Zealand/Australia.
Can you list a situation where nutrition may be a priority over other lines of therapy (e.g., pharmaceutical)?	Identify students’ ability to prioritise nutrition therapy in certain situations.
Where do you locate information about healthy eating? (probe) - What about a specific condition? (e.g., malnutrition or an inborn area of metabolism) - What would you do with this patient or where would you find information to provide?	Identify students’ ability to locate reputable sources of information for healthy eating and for specific conditions which require nutrition care.
Do you have any other comments that you would like to add?	Explore students’ perceptions related to medical nutrition education.
